# Genome-Wide Identification and Co-Expression Analysis of WRKY Genes Unveil Their Role in Regulating Anthocyanin Accumulation During *Euscaphis japonica* Fruit Maturation

**DOI:** 10.3390/biology14080958

**Published:** 2025-07-29

**Authors:** Bobin Liu, Qingying Wang, Dongmei He, Xiaqin Wang, Guiliang Xin, Xiaoxing Zou, Daizhen Zhang, Shuangquan Zou, Jiakai Liao

**Affiliations:** 1 Jiangsu Provincial Key Laboratory of Coastal Wetland Bioresources and Environmental Protection, Jiangsu Key Laboratory for Bioresources of Saline Soils, Jiangsu Synthetic Innovation Center for Coastal Bio-Agriculture, School of Wetlands, Yancheng Teachers University, Yancheng 224051, China; liubb@yctu.edu.cn (B.L.); xglnuw@163.com (G.X.); daizhen79wenxin@163.com (D.Z.); 2 College of Forestry, Fujian Colleges and Universities Engineering Research Institute of Conservation and Utilization of Natural Bioresources, Fujian Agriculture and Forestry University, Fuzhou 350002, Chinazxx0299@fafu.edu.cn (X.Z.); 3 Jiangsu Yancheng Coastal Wetland Ecosystem Positioning Observation and Research Station, Jiangsu Academy of Forestry, Nanjing 211153, China; 4 National Key Laboratory for Development and Utilization of Forest Food Resources, College of Forestry and Biotechnology, Zhejiang A&F University, Hangzhou 311300, China; 5 School of Data Science, Fuzhou University of International Studies and Trade, Fuzhou 350202, China; 6 Basic Forestry and Proteomics Research Center, School of Future Technology, Haixia Institute of Science and Technology, Fujian Agriculture and Forestry University, Fuzhou 350002, China

**Keywords:** *Euscaphis japonica*, WRKY, anthocyanins, genome-wide

## Abstract

Anthocyanins, crucial water-soluble pigments in plants, determine coloration in floral and fruit tissues, while fulfilling essential physiological roles in terms of plant growth, development, and stress adaptation. The biosynthesis of anthocyanins is transcriptionally regulated by WRKY factors, one of the largest plant-specific transcription factor families. *Euscaphis japonica* is an East Asian species, prized for its exceptionally persistent butterfly-shaped fruits that undergo pericarp dehiscence, overturning, and a color transition to scarlet red. This species represents an ideal system for studying anthocyanin regulation. However, the mechanisms by which WRKY transcription factors orchestrate anthocyanin accumulation during this process remain unknown. In this study, we identified 87 WRKY genes (*EjaWRKYs*) from the *E. japonica* genome. Phylogenetic analysis was used to classify these genes into three primary groups, with five subgroups, revealing conserved gene structures and motif compositions, supported by collinearity and comparative synteny analyses. Crucially, ten *EjaWRKYs* exhibited peak expression during the mature fruit stages, showing positive correlations with key anthocyanin biosynthesis genes. Functional validation through the use of transient transactivation assays in *Nicotiana benthamiana* confirmed that the five selected *EjaWRKYs* bind W-box elements and strongly activate reporter gene expression. Our results reveal *EjaWRKY*s’ regulation of anthocyanin accumulation in *E. japonica* fruit, provide the first comprehensive WRKY family characterization of this species, and establish a foundation for manipulating ornamental traits in horticultural breeding.

## Simple Summary

The *Euscaphis japonica* is prized for its unique butterfly-shaped fruits that maintain their bright red color for over half a year, much longer than most plants. In this study, we sought to understand how these trees maintain such lasting color. Our study discovered 87 special genes that act like color switches. Ten of these switches activate most strongly when the fruits become fully red, in concert with other color-making genes. Laboratory tests discovered that five key switches directly activate red pigment production. As the first exploration of these color-control genes in *E. japonica*, our findings help explain the extraordinary aesthetics of this fruit. This knowledge will help protect this rare tree and help design new ornamental plants with vibrant, long-lasting colors for future gardens.

## 1. Introduction

Anthocyanins represent a class of water-soluble flavonoid pigments, ubiquitously distributed across plant species. These compounds serve as primary determinants of coloration in floral organs, foliage, and fruits, through variations in their composition and concentration [1]. Beyond their aesthetic functions, anthocyanins play fundamental roles in plant physiology, contributing significantly to growth regulation, developmental processes, and adaptive responses to environmental challenges [2,3]. From a human perspective, anthocyanins offer valuable natural alternatives to synthetic food colorants, while conferring documented health benefits [4,5]. Therefore, due to the significance of anthocyanins as key secondary metabolites for both plants and humans, integrated research on the regulation of anthocyanin biosynthesis is essential.

Functionally, anthocyanins act as photoprotective agents in developing leaves, evidenced by their higher accumulation in juvenile foliage compared to that in mature tissues [6]. In reproductive structures, the intensity of flower and fruit coloration directly correlates with anthocyanin levels [7], enhancing pollen protection, attracting pollinators to ensure reproductive success [8], and facilitating seed dispersal by frugivores [9]. Notably, anthocyanin biosynthesis is highly responsive to abiotic stresses. Rapid anthocyanin accumulation occurs under conditions such as low temperature and high light intensity in species including apple (*Malus domestica*), pear (*Pyrus communis*), and lettuce (*Lactuca sativa*) [10,11,12]. This inducible synthesis is attributed to the potent antioxidant capacity of anthocyanins, which effectively scavenge reactive oxygen species generated during photosynthetic electron transport under stress [13]. Consequently, anthocyanin-mediated coloration is a major factor influencing the ornamental and horticultural value of plants.

The synthesis of anthocyanins is orchestrated by a complex transcriptional regulatory network that is responsive to environmental cues [14,15]. Among the key regulatory families, WRKY transcription factors constitute one of the largest plant-specific groups [16]. WRKY proteins are characterized by the highly conserved WRKYGQK amino acid sequence and C2H2/C2HC-type zinc finger motifs within their DNA-binding domain. These proteins specifically recognize the W-box cis-element (TTGACC/T) in target gene promoters [17,18]. Emerging evidence underscores the direct involvement of WRKY family members in anthocyanin regulation [19]. For instance, in red-fleshed apples, *MdWRKY11* modulates flavonoid and anthocyanin biosynthesis through interactions with *MdMYB* transcription factors and the photoresponse regulator, *MdHY5* [20]. Light-induced anthocyanin synthesis in pear involves *PpWRKY44*, which regulates *PpMYB10* expression [21]. Genome-wide characterization in *Populus* and functional studies in *Lycoris radiata* confirm WRKY’s involvement in anthocyanin biosynthesis under drought stress and methyl jasmonate treatment [22]. *PbWRKY75* promotes anthocyanin synthesis by activating key biosynthetic genes (*PbDFR*, *PbUFGT*) and the regulatory gene, *PbMYB10b*, in pear [23]. The heterologous expression of *Brassica napus* WRKY41-1 enhances anthocyanin accumulation in *Arabidopsis thaliana* [24]. Furthermore, a grapevine study demonstrated that the MYB5-driven MBW complex recruits a WRKY factor to enhance the expression of targets involved in vacuolar hyper-acidification and anthocyanin trafficking [25]. The importance of *FaWRKY71* in strawberry (*Fragaria × ananassa*) fruit ripening further highlights the conserved regulatory role of this family across diverse species [26]. Collectively, these studies establish WRKY transcription factors as integral components of the anthocyanin regulatory network.

The WRKY gene family was first identified with the discovery of SPF1 in sweet potato (*Ipomoea batatas*) [27]. Subsequently, *WRKY* families have been systematically characterized across diverse plant species, including Arabidopsis (*A*. *thaliana*; 72 genes) [28], rice (*Oryza sativa*; 100 genes) [28], pineapple (*Ananas comosus*; 54 genes) [29], and sunflower (*Helianthus annuus*; 90 genes) [30]. While WRKYs are established regulators of diverse plant processes, including growth, development, and stress responses [16,18], research into their specific roles in anthocyanin regulation, particularly within non-model species possessing unique ornamental or adaptive traits, remains significantly underdeveloped. Elucidating the precise mechanisms by which WRKY factors govern anthocyanin biosynthesis in these systems is, therefore, an important research objective.

*Euscaphis japonica* (Thunb.) Kanitz (Staphyleaceae), previously called *E. konishii* in earlier literature [31], is a deciduous tree or shrub, native to East Asia, prized for its exceptionally persistent bright red fruits, which remain visually striking for over six months [9]. During maturation, the fruit undergoes a developmental transition, wherein the pericarp dehisces and overturns, evolving from green to an intense scarlet hue, thereby forming a distinctive butterfly-shaped structure and revealing contrasting black seeds [31,32]. This extended display period and vivid coloration enhance its attraction to avian seed dispersers [32]. Our previous multi-omics study identified a key mechanism underlying these traits, i.e., phosphorylation modifications at specific residues (S21/S37 and S394/S429) within the light signaling components, EjPHYB and EjPHOT1, respectively [9]. These modifications trigger auxin accumulation and suppress ethylene biosynthesis, initiating the development of the long-lasting red fruit characteristic of this species [9]. However, the downstream transcriptional regulators, particularly WRKY factors, orchestrating anthocyanin accumulation during this process remain unknown.

Despite comprehensive genomic identification of WRKY families in diverse plants, a systematic analysis in this regard for *E. japonica* is lacking. Given the established role of WRKYs in anthocyanin regulation across plant species, this study focuses on characterizing the WRKY family in *E. japonica*. We identified 87 WRKY genes from the *E. japonica* genome and characterized their phylogenetic relationships, gene structures, chromosomal locations, and expression patterns. This foundational analysis provides essential insights for deciphering how WRKY transcription factors regulate the vivid red coloration of *E. japonica* fruits, enabling the development of future targeted breeding strategies for this trait.

## 2. Materials and Methods

### 2.1. Identification of WRKY Transcription Factors in E. japonica

WRKY family members were identified using the HMMER v3.0 package, with the WRKY domain (PF03106), from the protein families (Pfam) database [33,34]. Two iterative HMMER searches were performed against the *E*. *japonica* proteome. Initial candidates were validated through domain scanning via the CDD (Conserved Domain Database) [35], Pfam [34], and SMART tools [36]. Non-redundant sequences were retained after removing partial/false positive domains.

### 2.2. Phylogenetic Classification

Full-length WRKY protein sequences from *E*. *japonica* and *A*. *thaliana* were aligned using MUSCLE v3.8.31 [37]. A maximum-likelihood tree was constructed using IQ-TREE v2.1.2 and the JTT + G4 model, with branch support assessed using 1000 ultrafast bootstrap replicates [38]. The tree was visualized and annotated using EvolView v3.0 [39].

### 2.3. Gene Structure and Conserved Motif Characterization

All 87 EjaWRKY sequences were submitted into ExPASy (http://web.expasy.org/protparam/; accessed on 10 June 2021) for calculations, including the number of amino acids, molecular weight, and isoelectric points, etc., and default parameters were applied for all the calculations [40]. Conserved motifs in the EjaWRKY protein were identified using the MEME online program [41], with the search parameters allowing zero or one occurrence per sequence and a maximum of 10 motifs. We used TBtools v2.008 [42] to generate gene structure diagrams for the EjaWRKYs, thereby depicting their exon–intron organization, and to visualize both gene structures and conserved motifs.

### 2.4. Genomic Distribution and Duplication Events

Chromosomal positions were mapped using genome annotation data, visualized in TBtools v2.008 [42]. We identified tandem duplications when the genes met two criteria: (1) >75% sequence coverage of the longer gene and (2) >75% sequence similarity within 200 kb genomic regions containing ≤5 intervening genes. Segmental duplications and syntenic relationships with *Arabidopsis* were analyzed using MCScanX v1.0.0 [43] and visualized with Circos v0.69 [44]. We calculated the Ka/Ks ratios using TBtools v2.008 [42] to estimate the selection pressures.

### 2.5. Promoter Cis-Element Analysis

Promoter sequences were defined as the 2000 bp genomic region upstream of the 5′-UTR for each *EjaWRKY* gene. These sequences were analyzed using the PlantCARE database [45] to identify *cis*-regulatory elements. Following automated detection, the elements were manually curated to verify 12 functionally significant types: W-box (WRKY binding), the CGTCA-motif (MeJA response), MBS I (flavonoid biosynthesis), LTR (low-temperature response), TC-rich repeats (defense response), ABRE (ABA response), circadian elements, MBS (drought response), the WUN-motif (wound response), the TGA-element (auxin response), the GARE-motif (gibberellin response), and the TCA-element (salicylic acid response). Element distributions across all the *EjaWRKY* promoters were quantified and visualized using TBtools v2.008 [42].

### 2.6. Expression Profiling

The tissue-specific expression of *EjaWRKY* genes was quantified using Fragments Per Kilobase per Million mapped reads (FPKM) values, derived from publicly available RNA-seq datasets [31,32,46], and the detailed transcriptomic analysis has been described previously [32]. Heatmaps illustrating *EjaWRKY* expression patterns were generated using ClusterGVis v 0.1.1 [47].

### 2.7. Correlation Analysis and RT-qPCR Verification

Thirty-three key anthocyanin pathway genes that have been previously identified [48], including *EjaCHS1-9*, *EjaCHI1-3*, *EjaF3H1-4*, EjaF3′H, *EjaF3′5′H1-2*, *EjaDFR1-2*, *EjaLAR*, *EjaANS1-2*, *EjaANR1-2*, and *EjaUFGT1-7*, were analyzed for correlations with *EjaWRKYs* in R v4.0, using the psych package v2.1.3 [49], using FPKM values from multiple tissues (bra, lea, flo, peA, piA, SeaA, TFRA, and Fr_I-IV). Using Pearson’s correlation coefficients, with a significance threshold of *p* < 0.05, we selected relationships exhibiting a strong correlation (|*r*| = 0.68–0.90) or a very strong correlation (|*r*| = 0.91–1.00) for further investigation [50]. These results were visualized using Cytoscape v3.9.1 [51]. *E*. *japonica* fruits representing five developmental stages (20, 55, 75, 115, and 160 DAF) were harvested as described previously [9], corresponding, respectively, to green fruit (20 and 55 DAF), color-changing fruit (75 DAF), red fruit with a newly split pericarp (115 DAF), and fully split fruit (160 DAF). The total RNA extraction, reverse transcription, RT-qPCR, and relative expression level calculations followed established protocols [48], using the primers listed in Table S1.

### 2.8. DNA Binding and Transactivation Assay

Full-length *EjaWRKYs* were cloned into pGreenII62-SK to generate *35S::EjaWRKY* effectors, while the W-box *cis*-element was inserted into pGreenII 0800-LUC to construct the reporter. These constructs were transformed into the *Agrobacterium tumefaciens* strain, GV3101, for transient expression in *Nicotiana benthamiana*. Effector and reporter agrobacteria were co-infiltrated into the abaxial side of *N. benthamiana* leaves, using a needleless syringe. The relative LUC activity was measured 48–72 h post-infiltration, as previously described [52].

## 3. Results

### 3.1. Identification and Phylogenetic Analysis of the WRKY Family in E. japonica

**Figure 1:**
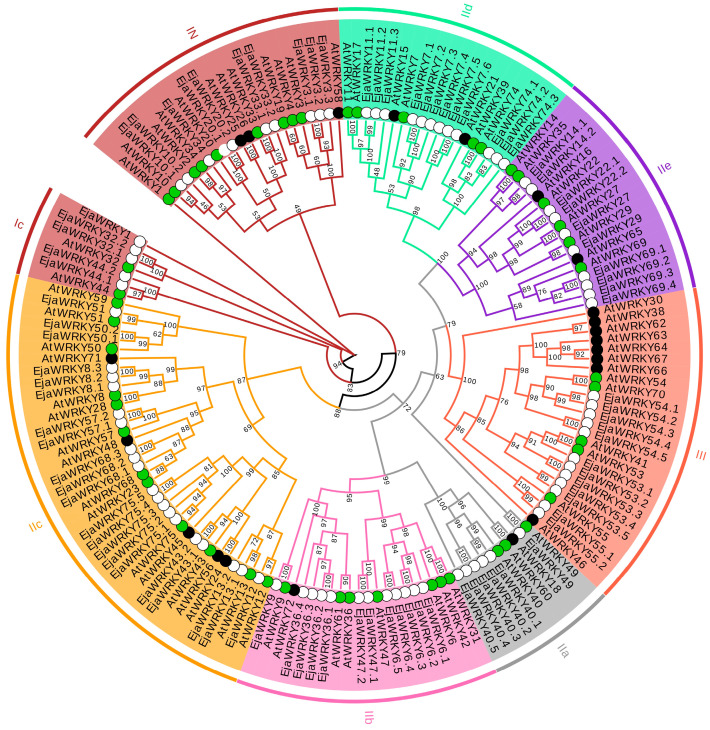
Evolutionary relationships of WRKY domains in *E. japonica* and *A. thaliana*. Unrooted phylogenetic tree of WRKY domains, with groups/subgroups color coded, as defined by Eulgem et al. [17]. Group I domains are subdivided into N-terminal (suffix ‘N’) and C-terminal (suffix ‘C’) clusters. *A. thaliana* and *E. japonica* domains are denoted by grass-green circles and hollow circles, respectively. Branch labels show bootstrap percentages (1000 replicates). Black solid circles indicate *Arabidopsis*-specific WRKYs lacking orthologs in *E. japonica*. The prefixes “At” (*A. thaliana*) and “Eja” (*E. japonica*) identify species origin.

To identify WRKY family members in *E. japonica*, we performed two rounds of HMMER v3.0 searches against the *E. japonica* proteome, using the WRKY domain (PF03106) as a query. The initial screening yielded 102 candidate proteins, which we used to build a species-specific HMM profile. Subsequent research identified 110 putative WRKY proteins. After removing redundant sequences, candidate proteins were validated via domain scanning (CDD, Pfam, SMART), yielding 87 non-redundant WRKY proteins after the removal of duplicates (Table S2). These genes were designated as EjaWRKYs based on phylogenetic clustering with *A. thaliana* orthologs (Table S2 and Figure 1). The protein characteristics, including the coding sequence (CDS) length, protein length, molecular weight (MW), isoelectric point (pI), corresponding gene’s chromosome localization, conserved motif, and zinc finger domain pattern are detailed in Table S3. EjaWRKY proteins ranged from 88 aa (EjaWRKY7.2; MW: 13.4 kDa) to 764 aa (EjaWRKY13.2; MW: 98 kDa), with pI values spanning 4.93 (EjaWRKY14.1) to 10.19 (EjaWRKY7.2) (Table S3).

A maximum-likelihood phylogenetic tree was constructed using the amino acid sequences of full-length WRKY protein sequences from *E. japonica* and *A. thaliana*. We classified EjaWRKYs into established groups based on both their topological positioning and established *Arabidopsis* WRKY classifications. The 160 WRKYs (87 EjaWRKYs, 72 AtWRKYs) were resolved into three major clades (Groups I–III), as defined by Eulgem et al. [17] (Figure 1). Group I contained 15 EjaWRKYs, subdivided into N-terminal (IN) and C-terminal (IC) clusters. Group II contained 61 EjaWRKYs (IIa:8, IIb:12, IIc:21, IId:12, IIe:8), while Group III contained 11 EjaWRKYs. Twenty-four *A. thaliana* WRKYs lacked *E. japonica* orthologs, while 19 EjaWRKYs expanded into paralog pairs. *WRKY7* and *WRKY75* underwent pronounced duplication (six and five homologs, respectively; Figure 1). These results indicate that the WRKY family in *E*. *japonica* retains the fundamental three-group structure (I–III), but shows gene loss/expansion in select clades compared with *A. thaliana*.

### 3.2. EjaWRKY Protein Motif Composition and Corresponding Gene Structure Analysis

**Figure 2:**
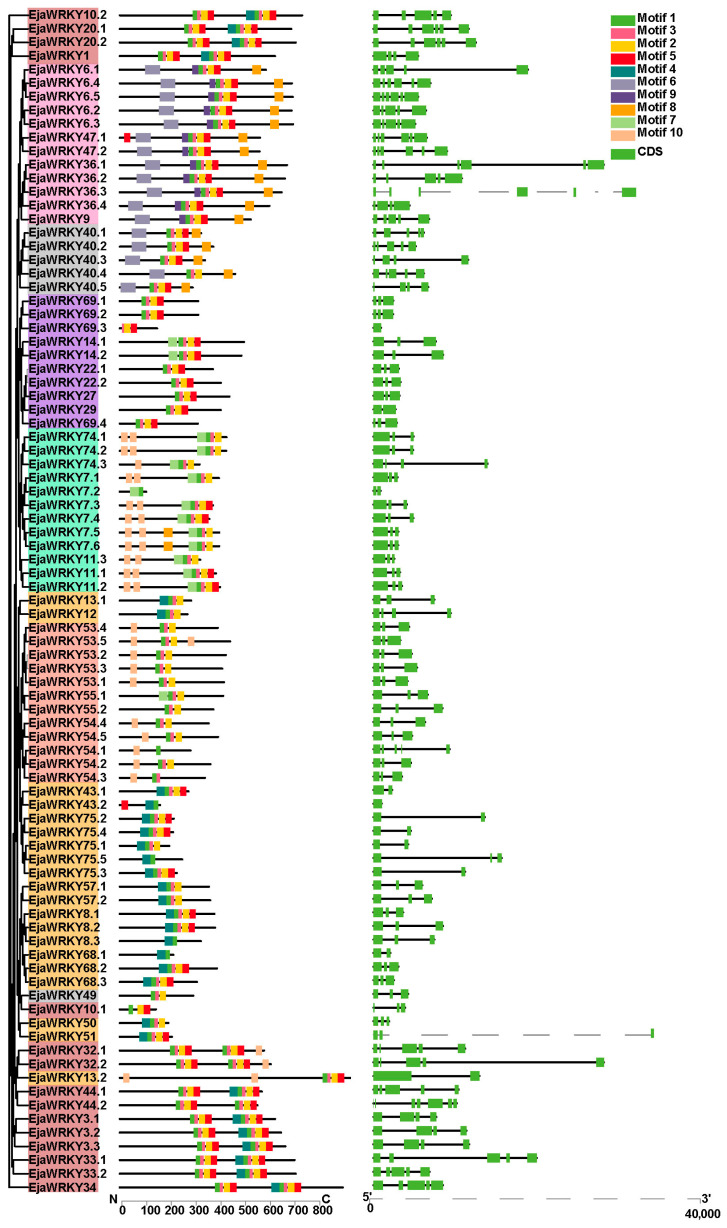
Phylogenetic relationships, protein domains, and gene structures of *E. japonica* WRKY proteins. An unrooted maximum-likelihood tree (left) clustering 87 WRKYs into color-coded phylogenetic groups, with adjacent schematics depicting conserved protein domains.

The conserved domains of EjaWRKYs were analyzed using MEME (https://meme-suite.org/meme/; accessed on 15 June 2021) to generate schematic representations of EjaWRKY family motifs (Figure 2 and Table S4). Motif 1, containing the signature WRKYGQK sequence characteristic of WRKY transcription factors, was universally conserved across all EjaWRKYs (Figure 2 and Table S3). Proteins within phylogenetic groups exhibited conserved motif composition and spatial arrangements (Figure 2). All Group I members (except for EjaWRKY10.1) contained two WRKYGQK domains (Figure 2 and Table S3). EjaWRKY50.1, EjaWRKY51, and EjaWRKY13.2 exhibited a single amino acid variation in Motif 1 relative to the canonical WRKY domains (Table S3), consistent with rice orthologs [28]. Subgroup IIb members (excluding EjaWRKY47.1) shared identical seven-motif architectures (Figure 2). Motifs 2, 3, and 5 collectively formed the zinc finger structure (C-X_7_-C-X_23_-H-X-C), absent in EjaWRKY7.2 and EjaWRKY54.1 (Table S3). Group III members uniformly retained one WRKYGQK domain and complete zinc finger motifs, except for EjaWRKY54.1 (Figure 2 and Table S3). Subgroup-specific motif distributions were then observed. We found that Motif 6 was localized exclusively to subgroups IIa/IIb, while Motif 9 occurred only in IIb (Figure 2). Paralogous gene clusters (e.g., EjaWRKY6.1–6.5, EjaWRKY36.1–36.4, and EjaWRKY57.1–57.2) maintained near-identical motif compositions (Figure 2). Notable domain differences in the proteins encoded by homolog pairs (EjaWRKY8.3/8.1, EjaWRKY69.3/69.1, EjaWRKY74.3/74.1, and EjaWRKY75.1/75.5) (Figure 2) suggest the occurrence of functional divergence.

The EjaWRKYs gene structure analysis revealed intron numbers ranging from 0 to 6. Two genes (2.3%) lacked introns, forty-two genes (47.7%) contained two introns, nine genes (10.2%) had one intron (including *EjaWRKY75.1–75.4*), and *EjaWRKY44.2* and *EjaWRKY50.2* contained six introns (the maximum observed) (Figure 2). Phylogenetically related genes showed conserved intron–exon organization (Figure 2). Twelve of the thirteen Group III members (excluding *EjaWRKY54.1*) shared two-intron structures (Figure 2). This structural conservation within clades validates the phylogenetic classification.

### 3.3. Chromosomal Organization of EjaWRKY Genes

**Figure 3:**
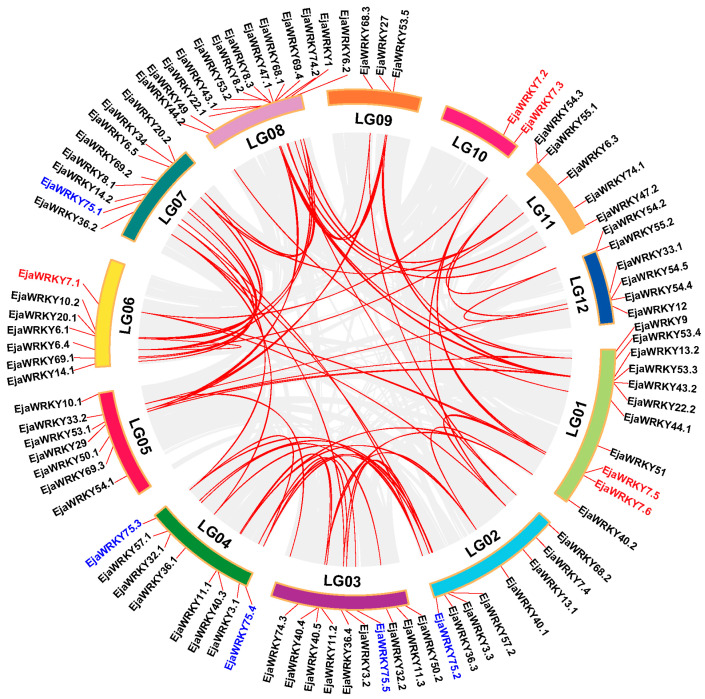
Chromosomal distribution and syntenic relationships of *E. japonica* WRKY genes. The 87 identified EjaWRKY genes are mapped across 12 linkage groups (LG01–LG12). Gene positions are marked with vertical ticks and labeled with gene names. Gray lines denote genome-wide synteny blocks; red lines highlight duplicated WRKY gene pairs (putative paralogs). Uneven gene density highlights chromosome-specific expansion biases (e.g., high density in LG07/LG08 vs. low density in LG09/LG10). Examples of putative segmental duplication events are highlighted in blue and red.

To elucidate the evolutionary trajectory of the WRKY transcription factor family in *E. japonica*, we performed a comprehensive collinearity analysis to map the genomic loci (Figure 3). The 87 identified EjaWRKY genes exhibited non-random chromosomal distribution across all 12 linkage groups (LGs), with significant clustering disparities observed (Figure 3). LG08 harbored the highest concentration (13 genes), contrasting sharply with LG10, which contained only two loci, while the remaining genes populated the other ten chromosomes (Figure 3). Notably, all *EjaWRKYs* resided within conserved syntenic blocks, exemplified by paralog clusters *EjaWRKY7.1–7.6* and *EjaWRKY75.1–75.5*, which arose from segmental duplication events (Figure 3). The absence of tandem duplications indicates that whole-genome duplication (WGD) mechanisms primarily drove the expansion of this gene family during *E. japonica* evolution. Selection pressure analysis of 73 duplicated gene pairs revealed constrained evolutionary rates, with nonsynonymous (Ka: 0.07–0.56) and synonymous (Ks: 0.31–3.67) substitution values yielding Ka/Ks ratios consistently below 1 (range: 0.07–0.61; Table S5). These uniformly low ratios reveal pervasive purifying selection acting on EjaWRKY genes, reflecting strong functional conservation and evolutionary constraint across duplicated paralogs.

### 3.4. Comparative Synteny Analysis of EjaWRKY Genes

**Figure 4:**
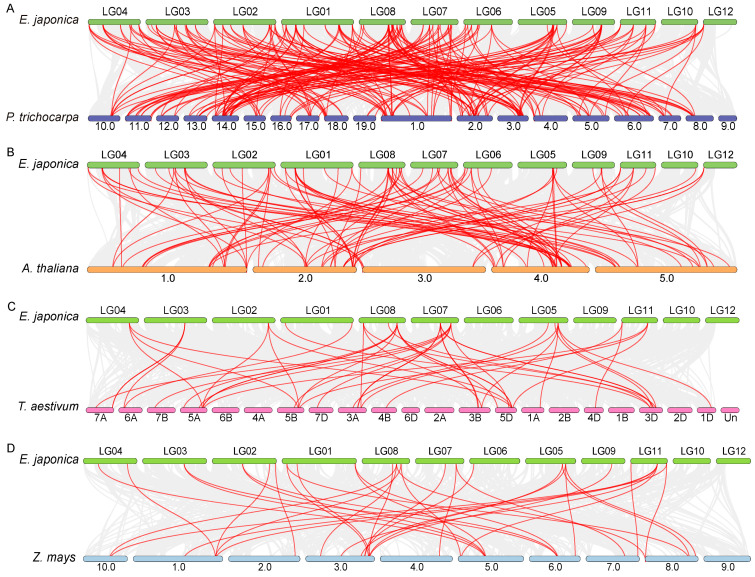
Synteny analysis of WRKY genes between *E. japonica* and four representative plant species. (**A**–**D**) Comparative genome collinearity analysis between *E. japonica* and (**A**) *P. trichocarpa*, (**B**) *A. thaliana*, (**C**) *Triticum aestivum*, and (**D**) *Zea mays*. Gray lines in the background indicate the collinear blocks within *E. japonica* and other plant genomes, while the red lines highlight the syntenic WRKY gene pairs.

To further determine the phylogenetic mechanisms shaping the WRKY gene family in *E. japonica*, we conducted a comprehensive comparative genome collinearity analysis between *E. japonica* and four representative plant species: the dicotyledonous models *A. thaliana* and *P. trichocarpa* and the monocotyledonous crops *Triticum aestivum* (wheat) and *Zea mays* (maize) (Figure 4). A total of 76 EjaWRKY genes exhibited syntenic relationships with orthologs in *P. trichocarpa* (Figure 4A). This number is comparable to the 64 syntenic orthologs identified with *A. thaliana* (Figure 4B). Strikingly, however, the number of syntenic *EjaWRKY* genes shared with the monocot species was substantially lower, with only 21 found in *Z. mays* and a mere 16 in *T. aestivum* (Figure 4C,D). This pronounced disparity underscores a closer evolutionary relationship between *E. japonica* (a rosid dicot) and its eudicot relatives (*A*. *thaliana*, *P*. *trichocarpa*) at the WRKY family level than with monocot representatives (*Z*. *mays*, *T*. *aestivum*), consistent with established angiosperm phylogeny [53].

Further evolutionary insights emerged from examining the specific gene pairs shared across lineages. We identified 41 syntenic WRKY gene pairs present in the dicot comparisons (*E. japonica–P. trichocarpa* and *E. japonica–A. thaliana*) that were entirely absent in the comparisons with the monocot species (*T. aestivum* and *Z. mays*) (Figure 4A,B). This absence strongly suggests that these 41 gene pairs represent lineage-specific collinear orthologs that originated after the evolutionary divergence of monocotyledonous and dicotyledonous plants. Conversely, the analysis uncovered 11 syntenic WRKY gene pairs that were conserved across all five species examined, *E. japonica*, *A. thaliana*, *P. trichocarpa*, *T. aestivum*, and *Z. mays* (Figure 4). The presence of these orthologous pairs in species spanning the fundamental monocot–dicot divide provides compelling evidence that such pairs reflect deeply conserved elements within the WRKY gene family. These 11 pairs are inferred to be ancient collinear orthologs that were already present in the genome of the last common ancestor of monocots and dicots and have been preserved through subsequent evolutionary radiation.

### 3.5. Cis-Regulatory Element Analysis of EjaWRKY Promoters

**Figure 5:**
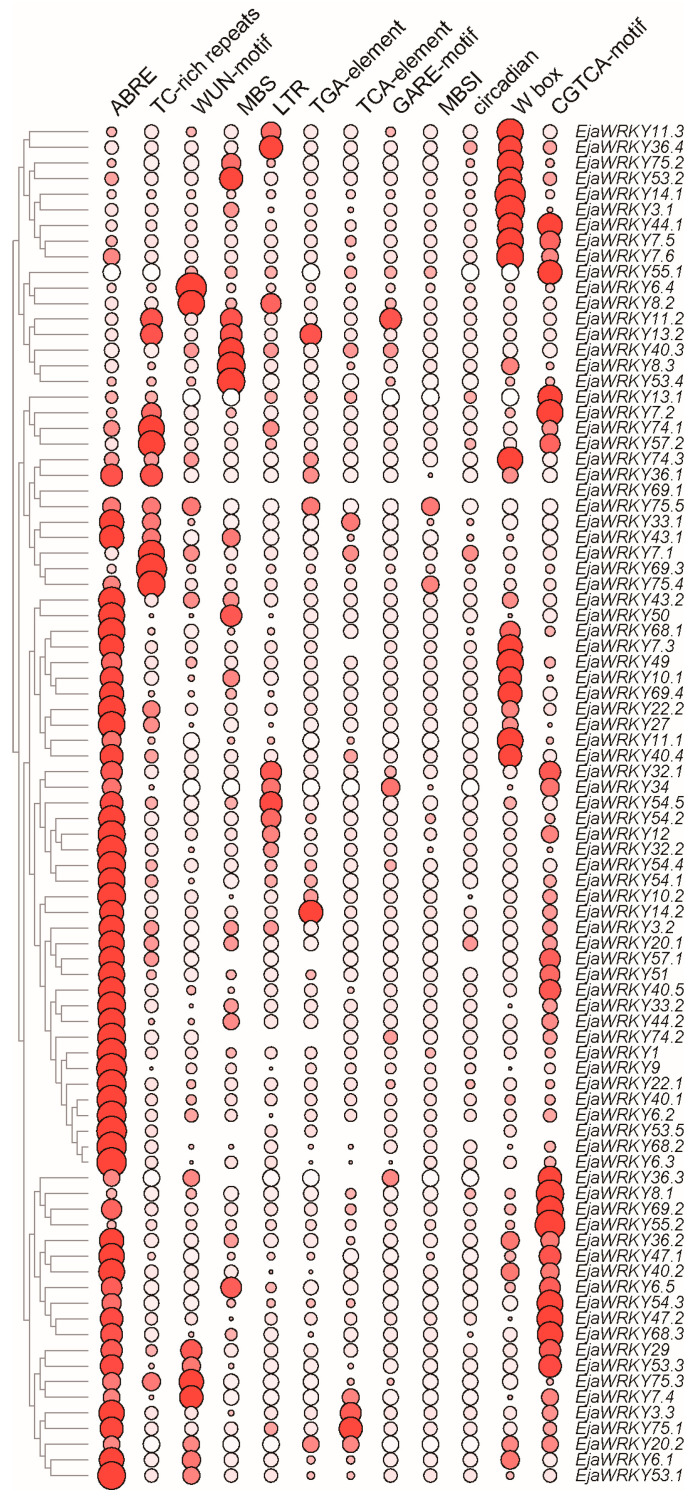
*Cis*-regulatory elements in *EjaWRKY* promoters. The distribution of 12 functionally annotated elements within 2000 bp upstream regions. Stress-responsive: LTR, MBS, WUN-motif, TC-rich repeats. Hormone-responsive: ABRE (ABA), CGTCA-motif (jasmonate), TGA-element (auxin), GARE-motif (gibberellin), and TCA-element (salicylic acid responsiveness). Developmental: MBS I (MYB site for flavonoid biosynthesis). TF binding: W-box (WRKY). The shade of color and the size of each circle both encode the predicted number of occurrences of a specific cis-regulatory element in the promoter region of a specific EjaWRKY gene.

Promoter analysis of *EjaWRKY* genes revealed abundant *cis*-regulatory elements associated with stress adaptation and hormonal signaling (Figure 5). We identified eight stress-responsive motifs, namely low-temperature (LTR), drought (MBS), wound (WUN-motif), and defense elements (TC-rich repeats), alongside four hormone-related elements, namely auxin (TGA-element), gibberellin (GARE-motif), ABA (ABRE), and jasmonate (CGTCA-motif). Notably, ABRE elements occurred in 92% of promoters, implicating ABA signaling as a central regulatory hub. The near-universal presence of W-box motifs (99% of promoters) suggests extensive autoregulation or co-regulation within the WRKY family. The element co-occurrence patterns indicate potential crosstalk between ABA, jasmonate, and stress-response pathways.

#### Spatial Expression Patterns of EjaWRKY Genes

**Figure 6:**
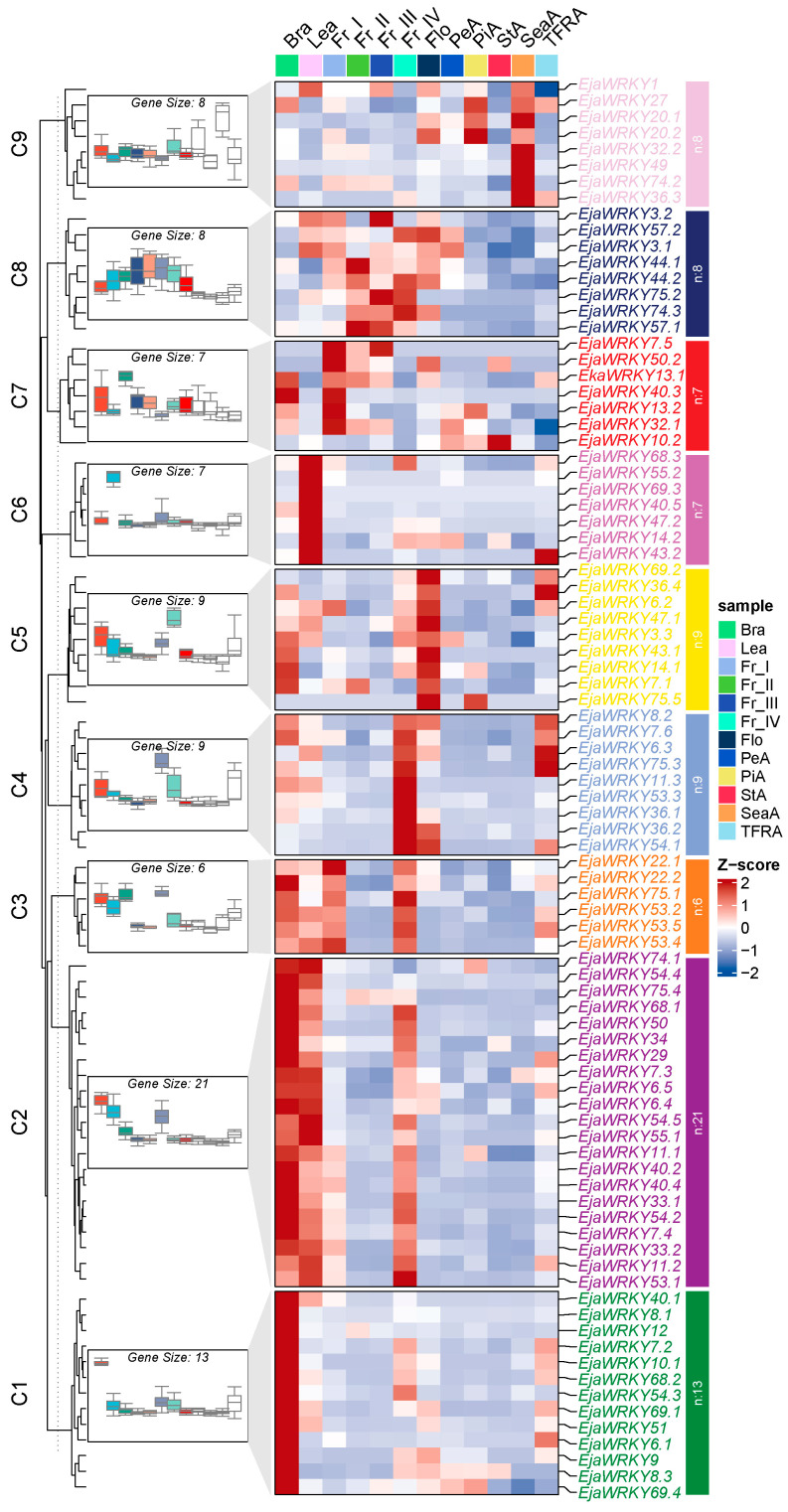
Expression profiles of the *E. japonica* WRKY genes. A Hierarchical clustering of the expression profiles of EjWRKY genes in 87 samples, including different tissues and developmental stages. The expression analysis included samples from multiple tissues: branch (bra), leaf (lea), flower (flo), petals (peA), pistil (piA), fully developed seeds (SeaA), and pseudoplump seeds (TFRA). Additionally, fruit samples were collected at four developmental stages: stage I (Fr_I, green fruit), stage II (Fr_II, color-changing fruit), stage III (Fr_III, newly split red fruit), and stage IV (Fr_IV, fully split fruit).

To investigate the potential physiological roles of *EjaWRKY* genes in *E. japonica* development, we analyzed publicly available RNA-seq data from branch (bra), leaf (lea), flower (flo), petals (peA), pistil (piA), fully developed seeds (SeaA), and pseudoplump seeds (TFRA), along with the fruit at four developmental stages, namely stage I (Fr_I, green fruit), stage II (Fr_II, color-changing fruit), stage III (Fr_III, newly split red fruit), and stage IV (Fr_IV, fully split fruit), representing the mature stage (Figure 6). All 87 EjaWRKY genes were categorized into nine distinct clusters based on their expression profiles, with each cluster exhibiting specific expression patterns (Figure 6). Cluster 1, which contains 13 EjaWRKY genes, showed high expression in branches, while Cluster 4 demonstrated fruit maturation stage (Fr_IV)-specific expression (Figure 6). Genes grouped in Cluster 5, 6, and 9 exhibited high transcription levels in flowers, leaves, and petals, respectively (Figure 6). Cluster 8 genes displayed dynamic expression during fruit development, as exemplified by *EjaWRKY74.3* and *EjaWRKY75.2*, whose mRNA abundance gradually increased (Figure 6). The paralogs *EjaWRKY36.1*–*36.4* exhibited divergent expression patterns: *EjaWRKY36.1* and *36.2* were expressed in fully split fruit (Cluster 4), *EjaWRKY36.3* in fully developed seeds (SeaA, Cluster 9), and *EjaWRKY36.4* was highly expressed in pseudoplump seed tissue (Cluster 5) (Figure 6). Similarly, *EjaWRKY75.1*–*75.3* was expressed in fully split fruit, while *EjaWRKY75.4* and *75.5* showed no detectable expression, with all five members grouped into different clusters (Figure 6). These results indicate that duplicated EjaWRKY genes do not necessarily retain similar expression patterns, suggesting possible neofunctionalization during evolution. The high expression observed in fully split fruit and flowers implies that cluster-specific EjaWRKY genes may regulate maturation processes in these reproductive tissues.

### 3.6. Association Analysis Between EjaWRKY Genes and Anthocyanin Biosynthesis

**Figure 7:**
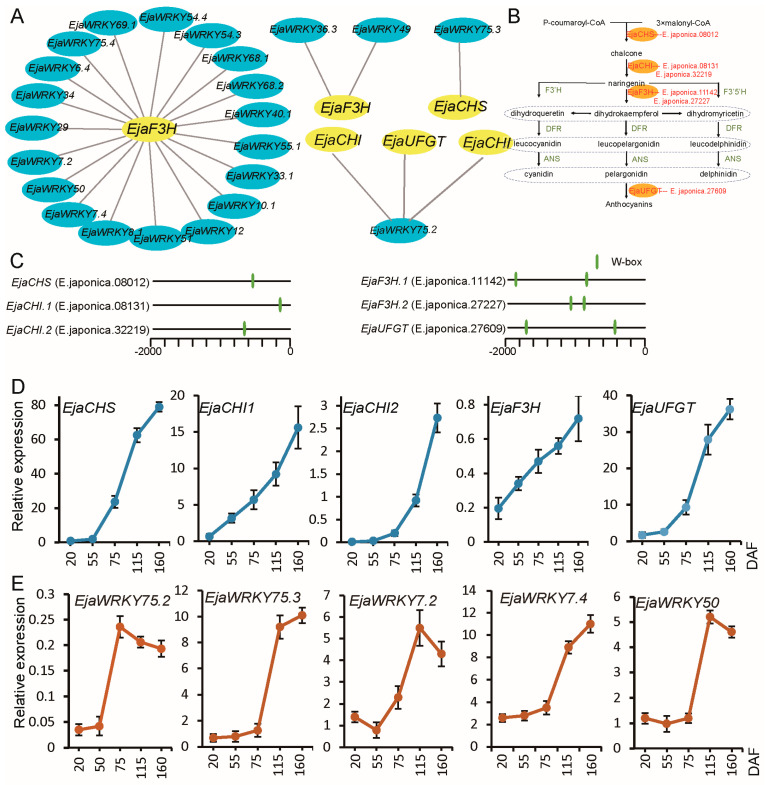
Integration of EjaWRKY regulatory networks with anthocyanin biosynthesis in *E. japonica*. (**A**) Correlation analysis (cor > 0.75) was performed between 87 *EjaWRKY* genes and 57 anthocyanin synthesis-related genes in *E. japonica*. Blue indicates *EjaWRKY*s; yellow represents anthocyanin synthesis-related genes. (**B**) Schematic diagram of the anthocyanin biosynthesis pathway. (**C**) Co-expressed anthocyanin synthesis pathway genes containing at least one WRKY binding site in their promoter region. (**D**,**E**) Expression dynamics of the co-expressed *EjaWRKY*s (**D**) and anthocyanin synthesis pathway genes (**E**) during *E. japonica* fruit maturation, analyzed by qPCR.

Previous transcriptome annotation studies have identified anthocyanin synthesis genes in *E. japonica* [48]. To investigate potential regulatory relationships between EjaWRKY transcription factors and anthocyanin biosynthesis, we performed correlation analyses involving 87 EjaWRKY genes and these anthocyanin pathway genes. The results demonstrated that 23 *EjaWRKYs* (26.4%) possessed significant positive correlations with anthocyanin biosynthesis genes under stringent thresholds, wherein the correlation coefficient exceeded 0.75, and the *p*-value was less than 0.01 (Figure 7A), suggesting their potential regulatory functions. Notably, specific anthocyanin pathway enzymes, including flavanone-3-hydroxylase (F3H) encoded by E. japonica.27227 and E. japonica.11142, chalcone synthase (CHS) encoded by E. japonica.08012, chalcone–flavanone isomerase (CHI) encoded by E. japonica.32219 and E. japonica.08131, and UDP-flavonoid glucosyltransferase (UFGT) encoded by E. japonica.27609, showed strong associations with *EjaWRKYs* (Figure 7B). Among the 23 correlated *EjaWRKYs*, all except *EjaWRKY75.2* and *EjaWRKY75.3* were associated with F3H-encoding genes.

Promoter analyses of six key anthocyanin biosynthesis genes revealed that all six aforementioned anthocyanin genes contained at least one WRKY-binding site (W-box). For example, F3H-encoding genes E. japonica.27227 and E. japonica.11142 contained one and two W-boxes, respectively (Figure 7C), suggesting potential regulatory mechanisms for EjaWRKY-mediated anthocyanin biosynthesis. To validate the co-expression patterns, we selected five EjaWRKY genes and five anthocyanin pathway genes for quantitative real-time PCR (qPCR) analysis during fruit development from 20–160 DAF, including green fruit (20 and 55 DAF), color-changing fruit (75 DAF), red fruit with a newly split pericarp (115 DAF), and fully split fruit (160 DAF). All anthocyanin-related genes exhibited progressive upregulation throughout fruit maturation (Figure 7D). In contrast, *EjaWRKYs* expression showed abrupt increases: *EjaWRKY75.2* and *EjaWRKY7.2* initiated substantial transcription at 50 DAF, while the other three genes were upregulated after 75 DAF. These dynamics suggest that EjaWRKYs may regulate anthocyanin biosynthesis during late fruit maturation in *E. japonica*.

**Figure 8:**
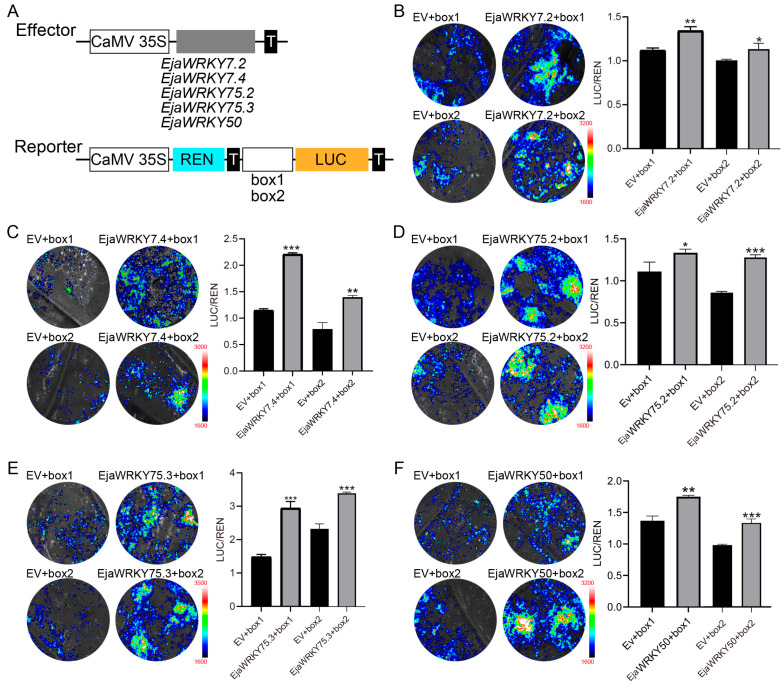
Functional validation of EjaWRKY transcription factors binding to W-box Cis-elements. (**A**) Schematic of effector (*35S::EjaWRKY*) and reporter (*W-box::LUC*) constructs for transient expression assays. (**B**–**F**) Luminescence imaging showing *W-box::LUC* activation in *Nicotiana benthamiana* leaves co-expressed with *35S::EjaWRKY7.2* (**B**), *35S::EjaWRKY7.4* (**C**), *35S::EjaWRKY75.2* (**D**), *35S::EjaWRKY75.3* (**E**), and *35S::EjaWRKY50* (**F**). EV represents empty vector. Data represent the mean ± SD; * *p* < 0.05, ** *p* < 0.01, *** *p* < 0.001 (*n* = 3 biological replicates; 3 technical replicates each).

To determine whether *E. japonica* WRKY transcription factors bind to anthocyanin biosynthesis genes and activate their expression, we cloned the W-box cis-element into a reporter vector to construct *W-box::LUC*, using *35S::REN* as an internal control (Figure 8A). The effector construct *35S::EjaWRKY* was co-expressed in *Nicotiana benthamiana* epidermal cells via transient expression assays. Negative controls included empty effector plus *W-box::LUC*. All five tested WRKYs significantly activated LUC expression (Figure 8B–F), demonstrating their capacity to bind W-box elements and transcriptionally activate downstream genes in planta.

## 4. Discussion

WRKY transcription factors constitute one of the largest and most extensively studied gene families in higher plants, playing crucial roles in diverse biological processes, including stress responses and development. Studies involving model species, such as Arabidopsis, rice, and sunflower, have provided foundational knowledge on this family. Within the *E. japonica* genome, we identified 87 WRKY genes (Figure 1; Table S2). This number places *E. japonica* within an intermediate range compared to other characterized species and is notably lower than counts in certain dicotyledonous species, such as soybean (*Glycine max*, 133 genes) [54] and poplar (*Populus euphratica*, 107 genes) [55]. However, this number is higher than that in cucumber (*Cucumis sativus*, 61 genes) [56] and carrot (*Daucus carota*, 67 genes) [57]. Similarly, compared to monocotyledons, the *E. japonica* WRKY complement is smaller than that of rice (*Oryza sativa*, 100 genes) [28] and maize (*Zea mays*, 128 genes) [58], but larger than that in pineapple (*Ananas comosus*, 54 genes) [29]. This intermediate gene count, which does not consistently align with either major angiosperm group, likely reflects the species-specific evolutionary history of *E. japonica*. Notably, the absence of detectable recent segmental duplication events may have constrained the expansion of the WRKY family (Figure 3). Furthermore, while some WRKY genes are located within regions derived from ancient whole-genome duplication events, potentially facilitating the expansion of specific subclades, comparative analysis with Arabidopsis suggests significant lineage-specific gene loss in certain subclades, possibly due to post-WGD evolutionary attrition or unidentified genomic events [59,60]. Critically, WRKY genes retained from ancient WGD events exhibit signatures of purifying selection (Figure 3 and Table S4), indicative of conserved functional importance. Collectively, these factors, namely limited recent duplication, lineage-specific retention/loss following ancient WGD, and functional constraints on retained duplicates, provide a plausible explanation for the observed size of the WRKY repertoire in *E. japonica*.

Syntenic analysis confirmed *E*. *japonica*’s closer evolutionary affinity to dicots than monocots (Figure 4). We identified 41 *EjaWRKY* gene pairs exclusively shared with dicot models (*P*. *trichocarpa*/*A*. *thaliana*). Conversely, only 11 syntenic pairs (e.g., *EjaWRKY57.1*, *EjaWRKY75.2*) were conserved across all five species (Figure 4), representing ancient orthologs, predating the monocot–dicot split. MEME analysis revealed near-universal conservation of the core WRKYGQK motif (Motif 1) across all 87 EjaWRKYs (Figure 2 and Table S3), despite its long evolutionary history, underscoring the critical functional importance of this DNA-binding domain. The transient expression of tobacco’s lower epidermis also indicates conserved DNA binding activity with W-box (Figure 8). The conserved gene structure patterns observed within clades provide further strong validation for this phylogenetic classification (Figure 1 and Figure 2). These results suggest that the WRKY family in *E. japonica* may have conserved biochemistry functions.

The red butterfly-shaped fruits of *E. japonica*, which are central to its ornamental and economic value, develop during summer months [9]. Our integrated analysis revealed that five EjaWRKY transcription factor family members exhibit significantly increased mRNA abundance after 55 DAF, maintaining elevated expression levels through the key anthocyanin accumulation stage at 115 DAF (Figure 7) [9,31]. Interestingly, the expression patterns of these five *EjaWRKYs* and core anthocyanin biosynthesis genes are not fully synchronized (Figure 7). This partial uncoupling suggests additional regulatory layers, potentially involving factors such as EjaARF5, which operates downstream of light-controlled auxin/ethylene signaling during ripening [9,48]. This convergence of EjaWRKY-mediated regulation with light-controlled auxin/ethylene signaling points to a sophisticated, coordinated network. Notably, fruit maturation coincides with intense summer heat, imposing significant abiotic stress. Given anthocyanins’ known role in scavenging reactive oxygen species and the conserved function of WRKYs in stress responses [13,16,18], the ripening-associated induction of EjaWRKYs likely serves a dual purpose: (1) coordinating anthocyanin biosynthesis for visual attraction and seed dispersal and (2) potentially activating downstream thermotolerance pathways to mitigate heat stress damage. The apparent coupling of pigmentation and stress adaptation through EjaWRKY induction represents an efficient evolutionary strategy, ensuring both fruit color and resilience under challenging field conditions. Consequently, targeting these EjaWRKY genes holds significant promise for breeding *E. japonica* varieties with enhanced ornamental appeal and climate resilience.

## 5. Conclusions

This study establishes *E. japonica* as a model for persistent pigmentation research by characterizing 87 WRKY transcription factors. The present results demonstrated lineage-specific evolution through limited recent duplications, while conserving the DNA-binding function. Crucially, ripening-induced EjaWRKYs were found to coordinate anthocyanin biosynthesis during scarlet fruit development, offering molecular targets for breeding superior horticultural varieties with increased visual appeal.

## Data Availability

All data generated or analyzed in this study are included in the main text and its Supplementary Materials.

## Supplementary Materials

The following supporting information can be downloaded at: https://www.mdpi.com/article/10.3390/biology14080958/s1, Table S1: The primers used in the study. Table S2: List of the 87 EjaWRKY genes identified in this study with associated sequences. Table S3: Characterization of the 87 EjaWRKY transcription factors identified in this study. Table S4: Conservative motifs of EkWRKY proteins. Table S5: Ka/Ks calculations of the duplicated EjaWRKY gene pairs in syntenic blocks.
